# Therapeutic potential of mesenchymal stem cell based therapy for osteoarthritis

**DOI:** 10.1186/s40169-016-0112-7

**Published:** 2016-08-10

**Authors:** John Burke, Monte Hunter, Ravindra Kolhe, Carlos Isales, Mark Hamrick, Sadanand Fulzele

**Affiliations:** 1 Department of Orthopedics, Georgia Regents University, Augusta, GA USA; 2 Department of Pathology, Georgia Regents University, Augusta, GA USA; 3 Department of Cell Biology and Anatomy, Georgia Regents University, Augusta, GA USA; 4 Institute of Regenerative and Reparative Medicine, Georgia Regents University, Augusta, GA USA; 5 Department of Orthopedics Surgery, Augusta University, Augusta, GA 30904 USA

**Keywords:** Osteoarthritis, Stem cell, ADSC, Cell therapy

## Abstract

Osteoarthritis (OA) is a chronic degenerative disease affecting articular cartilage in joints, and it is a leading cause of disability in the United States. Current pharmacological treatment strategies are ineffective to prevent the OA progression; however, cellular therapies have the potential to regenerate the lost cartilage, combat cartilage degeneration, provide pain relief, and improve patient mobility. One of the most promising sources of cellular regenerative medicine is from mesenchymal stem cells (MSCs). MSCs can be isolated from adipose tissue, bone marrow, synovial tissue, and other sources. The aim of this review is to compile recent advancement in cellular based therapy more specifically in relation to MSCs in the treatment of osteoarthritis.

## Introduction

Osteoarthritis (OA) is a chronic degenerative disease of articular cartilage that is the leading cause of joint disease in the United States. OA is characterized by the inability of chondrocytes to produce adequate functional matrix to compensate for matrix damage and depletion. Comorbidities such as aging, obesity, heart disease, diabetes, and mechanical stress become prevalent concerns in patients with osteoarthritis; in 2013, the center for disease control and prevention (CDC) found that 52.5 million adults over the age of 18 had self-reported physician-diagnosed arthritis, which is 22.7 % of the adult population [1, 2]. Treatment costs for solely knee OA are estimated to be $185.5 billion per year [3]. Conventional pharmacological interventions are not effective to prevent the OA progression. Recent advances in cell therapeutics offer potential methods to treat OA.

## Current therapies

OA is a chronic degenerative condition with no cure. Patients often experience pain, stiffness, swelling, loss of mobility, loss of flexibility, and weight gain secondary to reduced mobility/activity. Conventional OA therapeutics are directed toward symptomatic treatment, mainly pain management. Current treatment modalities for OA such as exercise, anti-inflammatory medication, and surgery are summarized below in Table 1. Current traditional therapies for OA have numerous downfalls in being perfect treatment strategies. Most importantly, these therapies fail to regenerate degenerated cartilages and prevent further degenerative processes. Recent advancements in molecular biology, regenerative, and reparative medicine offer new hope to develop novel therapeutic agents for OA like conditions.

**Table 1:** Current traditional pharmacological therapies for osteoarthritis

Treatment modality	Patient benefits	Shortcomings	References
Non-steroidal anti-inflammatory drugs (NSAIDs)	Analgesic effects; reduction in pain, stiffness, swelling	Often inadequate symptom relief; potential for liver damage in overdose; potential for ulcer and kidney disease; potential for bleeding and vascular events; can cause allergies; effectiveness is dependent upon patient compliance; will not reverse cartilage damage	[4–8]
Physical activity/therapy	Can improve flexibility, range of motion, and function of joint; can provide pain relief; strengthens muscles around the joint; targets obesity, the most important modifiable risk factor for OA	Often poor patient compliance; pain/symptom relief is often not enough for patient to adhere to the regiment; will not reverse cartilage damage	[9–12]
Opioids	Provide pain relief	Usefulness in the long-term is limited; Increased risk of adverse events (fractures, cardiovascular events, depression, addiction, overdose, mortality); numerous side effects; will not reverse cartilage damage	[9, 13–15]
Intra-articular injections	Hyaluronic acid injections can provide pain relief and improved function that can last over 8 weeks; corticosteroid injections can provide effective short-term pain relief and improved function	Injections must be performed in a doctor’s office; injections done more than once every 4 months can result in cartilage and joint damage and increase the risk of infection; hyaluronic acid injections show varying efficacy; neither type of injections will reverse cartilage damage	[9, 15–17]
Surgery	Total joint arthroplasty can potentially provide permanent pain relief and improved mobility; arthroscopic irrigation and debridement can offer pain relief	Many joints do not respond well to total joint arthroplasty; surgery is expensive for patients; increased risk of infection and invasive trauma; arthroscopic procedures do not provide long-term benefits	[9, 15, 17, 18]

## Cellular therapies

**Fig. 1:**
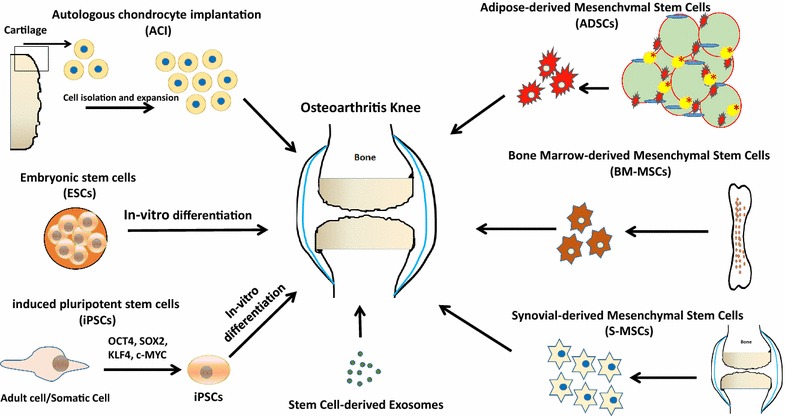
Schematic diagram illustrating the current clinical approaches to cell-based therapy for cartilage tissue engineering

Advancement in the field of cellular therapy for osteoarthritis is an exciting and quickly evolving area of research and medicine. Current cellular therapies are summarized in Table 2 and Fig. 1. One example of a cell based treatment that has improved over the past 20 years is a technique called autologous chondrocyte implantation (ACI). ACI is the only cellular based treatment with FDA approval and works by surgically obtaining autologous cartilage (i.e. the patient’s own cartilage) from a non-weight bearing area of the affected joint, isolating the chondrocytes via collagenase, expanding the chondrocytes in vitro, and finally injecting the cultured chondrocytes into the periosteum of the affected joint, with a graft to hold the cells in the desired location. [15, 19, 20]. The grafts that hold the cells in place have evolved from periosteal flaps and collagen I/III covered membranes to the latest method, matrix-induced ACI (MACI) [19]. While ACI has shown a success rate in patient improvement from 76 to 86 % (with Viste et al. showing the highest success), numerous problems and questions have been raised surrounding the procedure, including de-differentiation of the chondrocytes. ACI is also limited only the site of cartilage damage and not for generalized OA treatment [19–21].

**Table 2:** Therapeutic potentials and limitations of cell based therapies in osteoarthritis

Cellular therapy	Advantages	Shortcomings	References
Autologous chondrocyte implantation (ACI)	Success rate of 76–86 %; FDA approved; uses autologous cells; reduces pain; some instances of new production of durable cartilage-like tissue; MACI reduces graft hypertrophy	Requires two costly surgical procedures; less than 22 % chondrocytes successfully isolated; proliferation of chondrocytes decreases with patient age; chondrocytes often de-differentiate from type II collagen and lose capacity; specific ECM environment is required to keep chondrocytes differentiated, and ECM secretion capacity is lost with increasing chondrocyte age; periosteal grafts can detach, delaminate, and hypertrophy; uneven distribution of cells reported; often no clinical/radiographical changes	[9–24]
Embryonic stem cells (ESCs)	Can undergo self-renewal and are pluripotent; can differentiate into chondrocytes; unlimited proliferative potential; induce cartilage repair in animal models	Risk for teratoma formation, tumorgenicity, and immunogenicity; often require mouse fibroblasts to support cell growth, limiting human application; ESCs will likely never be used due to their ethically controversial nature; possibility of heterogeneous population of cells upon injection in patient; difficult to obtain large cell population of only chondrocytes	[15, 25–31]
Induced pluripotent stem cells (iPSCs)	Can undergo self-renewal and are pluripotent; unlimited proliferative potential; can differentiate into cartilage; can be derived from human chondrocytes and differentiated into chondrocytes; autologous source, which means immune rejection is less likely than in ESCs	Must be reprogrammed to differentiate into chondrocytes, which can be very difficult; risk for teratoma formation, tumorgenicity, and immunogenicity; difficult to obtain a uniform cell population; often obtain low yield of target cells; mechanisms poorly understood; cells retain epigenetic memory of original cell	[15, 25, 26, 29, 31–33]
Mesenchymal stem cells (MSCs)	Can undergo high rates of proliferation; can differentiate into chondrocytes; have immunosuppressive actions, privileged properties, anti-inflammatory effects, and pro-regenerative properties; have the ability to recruit other natural chondrocytes in patient tissue; very low risk for teratoma formation; well documented and studied; easy to isolate and expand; don’t require extensive reprogramming; cells are tissue specific and can come from a variety of autologous sources; have demonstrated significant cartilage improvement and patient improvement; can migrate to site of injury and release healing cytokines	Limited proliferation and differentiation potential in comparison to pluripotent stem cells; tissue specific morphology; their immunomodulative ability can cause problems; cells can be of heterogeneous population and difficult to classify; more randomized control trials are needed to better understand patient improvements	[15, 19, 20, 26, 31, 33–36]

Recent development in stem cell tissue engineering has created a lot of excitement in the field of cartilage regeneration biology. Stem cells are progenitor cells which differentiate into various cell types including osteoblasts, osteocytes, adipocytes, and cartilage [37–40]. Because of this, stem cells are being investigated for their abilities to regenerate cartilage in OA patients. These cells also have demonstrated the capability to inhibit T cell growth, thus showing that they have the ability to down-regulate the natural inflammatory response in OA [41]. While stem cells can both differentiate into new cartilage cells as well as suppress inflammation, recent studies have found that stem cells can also combat OA through paracrine mechanisms. They release important cytokines such as epidermal growth factor (EGF), transforming growth factor beta (TGFB), vascular endothelial growth factor (VEGF), as well as other cytokines and new cartilage proteins that are essential in combating OA and degenerative processes. It has also been suggested that stem cells could release cytokines and proteins that could help combat neurogenic pain, which would have numerous benefits in treating OA pain [40, 42]. Further research needs to be done in order to better understand stem cells mechanism of action in regard to their immunomodulatory, differentiating, paracrine, regenerative, and anti-inflammatory abilities as well as their cellular trafficking mechanisms.

Two types of stem cells being investigated are embryonic stem cells (ESCs, captured from embryonic mammalian cells) and induced pluripotent stem cells (iPSCs). Both cells possess the pluripotent ability to differentiate into chondrocytes or any type of cell; ESCs have been found to improve cartilage repair in animal models, and Wei et al. have generated iPSCs from human OA chondrocytes and subsequently induced the cells into chondrocytic differentiation [15, 25]. While there is some promise for both ESCs and iPSCs to differentiate into human cartilage to treat OA, many problems exist such as both cell types tend to cause teratoma growth as well as immunogenicity [26].

The other type of stem cell currently being investigated for its ability to treat OA is the mesenchymal stem cell (MSC). MSCs are a heterogeneous group of stromal cells that can come from a variety of sources including adipose tissue, bone marrow, and synovium; numerous studies have proven MSCs’ abilities to differentiate into chondrocytes and regeneratively treat OA [20]. While MSCs have a limited proliferative potential in comparison to pluripotent stem cells, many advantages exist for MSC based therapies [31]. MSCs and iPSCs offer the most realistic and best potential for viable regenerative cell treatment of OA; however, MSC based therapies have less risks associated with them and an easier means of production. The source of MSCs for treatment of OA is an important factor in cartilage tissue engineering and each cell type has its pros and cons (Table 2).

## Adipose-derived Mesenchymal Stem Cells (ADSCs)

Adipose-derived mesenchymal stem cells (ADSCs) can be harvested from the patient’s own adipose tissue most commonly via surgical resection or liposuction; specifically, infrapatellar fat pads (IFPs) provide cells with higher chondrogenic potential in comparison to other sources [43]. ADSC therapy for OA in animal studies is well documented, and the process of isolating ADSCs is not overly invasive [19]. Toghraie et al. demonstrated that ADSCs derived from IFPs in rabbits given to rabbits with OA-induced knees had less cartilage destruction, less subchondral sclerosis, less osteophyte buildup, as well as better cartilage overall than the control group [43]. Desando et al. also demonstrated that autologous ADSC therapy decreased the progression of degeneration in cartilage and in the synovial membrane. Furthermore, autologous ADSC therapy improved meniscal repair. Desando et al. suggested these findings could be due to the release of growth factors and cytokines. The regenerative effect of autologous ADSCs is dose and time dependent [44]. Another group also reported cartilage regeneration following autologous ADSC therapy in a surgically induced osteoarthritic sheep model. Autologous ADSCs were labeled and intra-articularly injected, leading to the cells populating the area of damaged cartilage as well as a decreased progression of OA [45]. While these animal models showed the promise of ADSCs in OA therapy, more studies like these need to be done in order to better understand the mechanisms so that they can be practiced in a routine clinical setting.

Several clinical studies also prove ADSCs’ efficacy in treating OA in human patients. Koh et al. treated patients with knee OA undergoing arthroscopic debridement with injected autologous ADSCs derived from IFPs and prepared in platelet rich plasma (PRP). Treated patients demonstrated improved mobility and function in the affected knees, reduced pain levels, and better clinical prognoses in a 1 year follow up [46]. In a 2 year follow up, Koh et al. found the patients had significantly improved Western Ontario and McMaster Universities Osteoarthritis (WOMAC) pain scores, VAS pain scores, and cartilage regeneration as confirmed by MRI [47]. This study suggests that these intra-articular injections are safe, and more clinical trials like these should be done to improve surgical and clinical outcomes. In a different proof-of-concept clinical trial, autologous ADSCs were injected in patients with knee OA. The high dose injection group had increased WOMAC scores 6 months after injections, decreased cartilage defects in affected areas, and increased cartilage volumes with thick, hyaline cartilage-like regeneration [48]. These results further proved the promising efficacy of autologous ADSCs in treating OA in humans, as well as demonstrated its safety as there were no adverse events. In 2011, Pak demonstrated the potential of ADSCs in osteonecrosis of the hip and OA in the knees of several patients. Results revealed bone formation in the osteonecrosis patients as well as cartilage regeneration in the OA knee patients. These patients’ MRIs had increased meniscus cartilage volume and thickness due to the ADSCs injections [49]. More similar clinical trials are necessary to further prove ADSCs’ efficacy and safety for routine use in the human OA setting.

## Bone marrow-derived mesenchymal stem cells (BM-MSCs)

Another source of MSCs for the treatment of OA is bone marrow-derived MSCs (BM-MSCs). BM-MSCs have a higher chondrogenic capability than ADSCs [50], and they have been studied more extensively than ADSCs [19]. Numerous animal models have demonstrated the potential therapeutic value of BM-MSCs, including one by Chiang et al. in 2016. They used allogenic BM-MSCs in combination with hyaluronic acid to treat knee OA-in a rabbit model, with the contralateral osteoarthritic knee only receiving hyaluronic acid. These treated rabbits were compared to untreated OA-induced rabbits. The joints treated with BM-MSCs and hyaluronic acid underwent less cartilage loss, fewer surface abrasions, and improved cartilage content [51]. This study showed that allogenic BM-MSCs can reduce the progression of OA. In a sheep OA model, autologous BM-MSCs were intra-articularly injected into the knees of sheep with arthroscopically-caused medial femorotibial condylar and meniscal defects. In comparison to the control group, the treated sheep showed signs of regeneration in their articular cartilage and menisci. The treatment group exhibited signs of statistically significant improvement in respect to both microscopic and histological guidelines [52]. These animal studies demonstrate the capability of BM-MSCs to combat OA.

In a human trial, Orozco et al. used autologous BM-MSCs to treat OA knee patients who were unresponsive to conservative treatments. BM-MSCs were intra-articularly injected, and their results indicated strong clinical efficacies such as improved cartilage quality in T2 mapping in 11 of the 12 patients and pain relief without hospitalization or surgery [53]. These findings suggest that BM-MSCs can be safely implemented in treatment strategies for treatment-resistant OA patients. Another study tested the efficacy of autologous BM-MSCs in treating patients with knee, hip, or ankle OA. Each patient received one autologous BM-MSC injection after the cells were isolated and cultured, and they were followed for 30 months. All patients enjoyed increased walking distances, improved WOMAC scores, and improved cartilage regeneration as demonstrated on MRI [54]. This study once again presented the regenerative potential of BM-MSCs in OA joints with minimal side effects. A randomized control trial in 2015 demonstrated the efficacy of allogenic BM-MSCs in treating knee OA. The study treated OA patients who had chronic knee pain and were unresponsive to conservative OA treatments with intra-articularly injected BM-MSCs in comparison to the control group who only received intra-articularly injected hyaluronic acid. The treatment group demonstrated decreases in poor cartilage areas, improved cartilage quality, and pain relief [55]. These findings are promising because it demonstrates BM-MSCs’ ability to inhibit the progression of OA; however, more clinical and basic science research, including human trials, must be done to understand molecular mechanisms and how the cells can better prevent the progression of OA in humans. Also, more studies must be done in order to effectively compare the chondrogenic abilities of different classes of stem cells (i.e. ADSCs and BM-MSCs) [56–59].

## Synovial-derived mesenchymal stem cells (S-MSCs)

Several human studies have been conducted on ADSCs and BM-MSC treatments for OA, but less has been done in recent times with synovial-derived MSCs (S-MSCs). In a rat knee OA model, S-MSCs injected weekly, rather than at a single time, were found to have migrated into the synovium and retained their undifferentiated S-MSC properties. The S-MSCs increased genetic expression of chondroprotective proteins such as BMP-2 and an anti-inflammatory gene, TSG-6 [60]. This suggests that periodic injections of S-MSCs can allow the MSCs to retain their MSC properties as well as inhibit the advancement of OA through genetic transcription. In a microminipig model, S-MSCs demonstrated the ability to enhance repair of longitudinally torn menisci in avascular areas. The group treated with S-MSCs had significantly improved meniscal healing at 12 weeks in comparison to the control group macroscopically, histologically, and by T1rho mapping [61]. In 2014, Hatsushika et al. further demonstrated the potential of S-MSCs by intra-articularly injecting them into the knees of pigs that had underwent medial meniscal resections. These damaged menisci regenerated cartilage significantly better than the control group in respect to MRI and histology. The treatment group’s cartilage was better preserved, and new synovial tissue filled the area of meniscal resection at 2 weeks [62]. This study, along with the previously mentioned studies, demonstrate that S-MSCs can provide a real answer in preventing the progression of OA as well as promoting regeneration of cartilage.

## Stem cell-derived exosomes

Although MSCs have demonstrated the unique ability and promise to combat degenerative diseases such as OA, they possess another mechanism of regenerative abilities not previously discussed: their exosomal products. Exosomes are packaged microvesicles that can contain proteins, lipids, factors, and/or genetic material that can be released in times of cellular stress [63, 64]. Furthermore, recent studies demonstrated transfer of genetic (miRNA, mRNA) material and protein through exosomal machinery [64, 65]. Large scale exosomes can be produced in the laboratory from stem cells and these exosomes can be used to combat the disease progression [64, 66]. If MSCs can be grown in culture and an environment similar to one of a chondrocyte undergoing osteoarthritic changes (via osteoarthritic cytokines), then the MSCs will release chondroprotective exosomes in response to the stress; these exosomal products could then be screened for, packaged in exosomes, and given to OA patients to promote cartilage regeneration [64]. Some miRNAs have already been identified in being involved in chondrogenesis and cartilage degeneration, including miRNA-101, miRNA-140, and miRNA 455 [67, 68]. Xu et al. found that human BM-MSCs release exosomes containing miRNAs that upregulate the Wnt pathway, leading to osteogenic differentiation [69]. These exosomal products can help explain how MSCs perform their regenerative abilities in combatting OA in a paracrine fashion, although it is impossible to say at this time how much of the healing is solely due to exosomes, and they may only offer a one-time relief instead of continuous relief that MSC-based treatment has demonstrated. More research is required to better understand how MSC-derived exosomal products mechanistically work, how they can be identified, and how they can be produced in Good Manufacturing Practices (GMP) in order to prevent the progression of OA in clinical medicine.

## Conclusions

Current treatment strategies for OA are inadequate and costly. Due to the increasing incidence and prevalence of OA, more innovative and effective therapeutic modalities need to be investigated, including MSCs. More randomized clinical trials need to be completed in order to demonstrate the efficacy, safety, and benefits of MSCs in treating patients with OA. Most of MSC research on humans only involves knee OA, and additional analysis should include clinical trials for ankle OA, shoulder OA, hip OA, and elbow OA. MSC-based cellular therapy has the potential and opportunity to effectively combat OA, but more extensive clinical trial and animal studies are required to understand the basic molecular mechanisms of MSC dependent cartilage regeneration. Further research is also necessary to better understand the potential of MSC-derived exosomes in the treatment of OA.
